# The Potential Diagnostic Biomarkers for the IgG Subclass in Coal Workers' Pneumoconiosis

**DOI:** 10.1155/2023/9233386

**Published:** 2023-03-14

**Authors:** Zhao-Guo Li, Bai-Cun Li, Zhi-Wei Li, Hui-Yuan Hu, Xia Ma, Hong Cao, Zhi-Hua Yu, Hua-Ping Dai, Jing Wang, Chen Wang

**Affiliations:** ^1^Department of Respiratory, The Second Affiliated Hospital of Harbin Medical University, Harbin, 150086 Heilongjiang, China; ^2^Institute of Basic Medical Sciences Chinese Academy of Medical Sciences, School of Basic Medicine Peking Union Medical College, Beijing 100730, China; ^3^First Clinical College, Xi'an Jiaotong University, Xi'an 710061, China; ^4^Department of Pulmonary and Critical Care Medicine, General Hospital of Datong Coal Mine Group Co., Ltd, Datong 037000, China; ^5^Department of Pulmonary and Critical Care Medicine, The First Hospital of Shanxi Medical University, Taiyuan 030001, China; ^6^Occupational Disease Prevention and treatment Hospital of Datong Coal Mine Group Co., Ltd, Datong 037001, China; ^7^Department of Pulmonary and Critical Care Medicine, Center of Respiratory Medicine, China–Japan Friendship Hospital, National Center for Respiratory Medicine, Institute of Respiratory Medicine, Chinese Academy of Medical Sciences, National Clinical Research Center for Respiratory Disease, Beijing 100029, China; ^8^National Center for Respiratory Medicine; Institute of Respiratory Medicine, Chinese Academy of Medical Sciences, National Clinical Research Center for Respiratory Diseases, Beijing 100029, China

## Abstract

Evidence suggests that exposure to coal dust increases immunoglobulin concentration. However, there is a paucity of data reporting immunoglobulin G (IgG) subclass in coal workers' pneumoconiosis (CWP). Therefore, this study intended to evaluate potential diagnostic biomarkers for the disease. CWP patients, dust-exposed workers without pneumoconiosis (DEW), and matched healthy controls (HCs) presented to the General Hospital of Datong Coal Mining Group and Occupational Disease Prevention and Treatment Hospital of Datong Coal Mining Group between May 2019 and September 2019 were recruited. The serum immunoglobulin concentration was determined by the multiplex immunoassay technique. Totally, 104 CWP patients, 109 DEWs, and 74 HCs were enrolled. Serum levels of IgG1, IgG2, IgM, and IgA were elevated in CWPs compared with those in DEWs and HCs (*P* < 0.05). The order of diagnostic accuracy between CWPs and DEWs depicted by the receiver operating characteristic (ROC) curve was IgG2, IgM, IgG1, IgG3, and IgA. Significantly higher IgG1/IgG3 and IgG2/IgG3 ratios were observed in the CWP group than in DEW and HC groups. Based on the IgG2/IgG3 ratio, the area under the ROC curve between CWP and DEW was 0.785 (95% CI 0.723-0.838), with a sensitivity of 73.1% and a specificity of 73.4%. Our findings suggest that IgG1, IgG2, IgM, and IgA are higher in the CWPs than DEWs and HCs. The IgG2/IgG3 ratio provides a viable alternative for the diagnosis of CWP.

## 1. Introduction

CWP, characterized by pulmonary inflammation and progression of fibrosis, is a chronic occupational lung disease caused by long-term inhalation of dust in the workplace [1]. Despite implementations of preventive measures for decades, the prevalence of CWP remains high across the world [2, 3]. Regular medical examinations for CWP mostly include exposure questionnaires, regular spirometry tests, and chest radiography. However, given the insidious early symptoms of CWP, early diagnosis and treatment are essential for disease prevention.

B-cell-derived immunoglobulins are routinely tested for CWP diagnosis in clinical practice as they provide critical information on humoral immunity status. Previous studies have shown increased IgA and IgG concentrations in CWP patients [4, 5]. However, it has been reported that immunoglobulins varied with the ethnicity in which immunoglobulins were performed [6, 7]. To date, IgG subclasses are frequently used as biomarkers in diagnosing certain diseases [8–10]. However, little knowledge is available related to the role of serum IgG subclass in the diagnosis of CWP.

We speculated that the ratio of IgG2 to IgG3 may be a viable indicator to distinguish CWPs from coal dust-exposed workers. To this end, the current study was performed to evaluate the diagnostic value of IgG2/IgG3 ratio for CWP.

## 2. Materials and Methods

### 2.1. Study Design

This study is a clinical diagnostic single-sample trial to evaluate the diagnostic value of IgG2/IgG3 ratio in DEWs. The sensitivity and specificity of IgG2/IgG3 were 0.7, *α* = 0.05 (unilateral), *β* = 0.1, and the ratio between groups was 1 : 1. The sample size was estimated to be at least 106 by PASS11, including 53 patients and 53 controls. Patients with CWP, DEWs, and healthy controls (HCs) admitted to the General Hospital of Datong Coal Mining Group and Occupational Disease Prevention and Treatment Hospital of Datong Coal Mining Group for CWP between May 2019 and September 2019 were recruited in this study, including 104 CWP patients and 109 DEWs. 74 cases of age and sex matched healthy subjects were selected as HCs. This research was approved by the Institutional Review Boards of Institute of Basic Medical Sciences, Chinese Academy of Medical Sciences, and General Hospital of Datong Coal Mining Group (No. 2018-DC125).

### 2.2. Inclusion and Exclusion Criteria

Inclusion criteria are as follows: CWP was diagnosed according to the China National Diagnostic Criteria for Pneumoconiosis [11].

Exclusion criteria are as follows: patients with bronchial asthma, active tuberculosis, diabetes mellitus, cardiovascular diseases, new inflammatory diseases, autoimmune disease, severe liver or kidney dysfunction, malignant tumors, previous treatment with drugs that interfere with the immune system (immunosuppressive or immunomodulator), glucocorticoids, previous radiotherapy, and anti-inflammatory drug usage within 1 month.

### 2.3. Spirometry Tests

Spirometry tests were performed according to the guidelines of ATS/ERS (2005) [12], and the parameters included forced vital capacity (FVC, %), forced expiratory volume in the first second (FEV1, %), and FEV1/FVC.

### 2.4. Serum Sample Collection and Immunoglobulin Measurement

5 mL peripheral blood samples were obtained from each participant following liver function test (within 2 hours). These blood samples were centrifuged at 3000 rpm for 10 min at 4°C to separate the serum, which was stored at -80°C for assay. The concentration of immunoglobulins (IgA, IgM, IgG1, IgG2, IgG3, and IgG4) was determined via Procarta Plex Multiplex immunoassay (MultiSciences Biotech, China) in strict accordance with the manufacturer's instructions.

### 2.5. Statistical Analysis

Statistical analysis was performed using the SPSS software version 20.0 (IBM, Armonk, NY, USA). Coherence to normal distribution analysis was conducted using the Shapiro-Wilk test. Normally distributed data were expressed as the mean ± SD, while the nonnormally distributed data were expressed as the median with interquartile range (IQR). Differences among the three groups (CWP, DEWs, and HCs) were examined using one-way analysis of variance (ANOVA) or the Kruskal–Wallis test. The Mann–Whitney *U* test was performed to analyze differences in abnormal distribution parameters between the two groups. Counting data was analyzed by the chi-square test. Logistic regression models were used to investigate the association between immunoglobulins and pneumoconiosis. The receiver operating characteristic (ROC) curve was plotted to determine the optimal threshold, sensitivity, specificity, and area under the curve (AUC) of each parameter between CWP and DEWs. In this study, the outcome was defined as the biomarker that provided the largest AUC of ROC (AUROC). The Delong test was used to compare AUC. Statistical significance was determined at *P* < 0.05.

## 3. Results

### 3.1. Participant Characteristics

There was no significant difference in age, BMI, hypertension status, and smoking status among the three groups. The CWP group drank less alcohol than DEWs and HCs (*P* = 0.022). The duration of exposure between the cases and dust-exposed worker was comparable (*P* > 0.05). The CWP group showed significant pulmonary function decline compared to DEWs and HCs, including the FEV1/FVC ratio, the predicted percentages of FVC and FEV1 (*P* < 0.001). Among the three groups, the number of white blood cells and neutrophils was lowest in the DEW group while lymphocytes were lowest in the CWP group (*P* = 0.031, *P* = 0.022, and *P* = 0.005, respectively) (Table 1).

### 3.2. Serum Concentrations of Immunoglobulins

**Figure 1:**
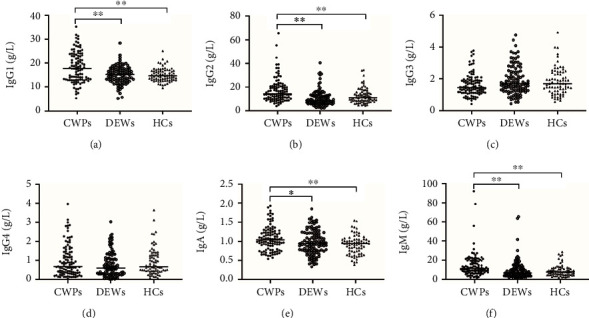
Comparison of serum concentrations of IgG1, IgG2, IgG3, IgG4, IgA, and IgM among the study groups. (a) IgG1, (b) IgG2, (c) IgG3, (d) IgG4, (e) IgA, (f) IgM. ^∗^*P* < 0.05; ^∗∗^*P* <0.01. CWP: coal workers' pneumoconiosis; DEWs: dust-exposed workers; HCs: healthy controls.

The serum IgG1, IgG2, IgA, and IgM concentrations were highest in CWP patients and lowest in HCs (*P* < 0.05). All the other indicators were comparable between groups (Figure 1).

### 3.3. Contribution of Serum Immunoglobulins Levels in Discriminating CWP

**Figure 2:**
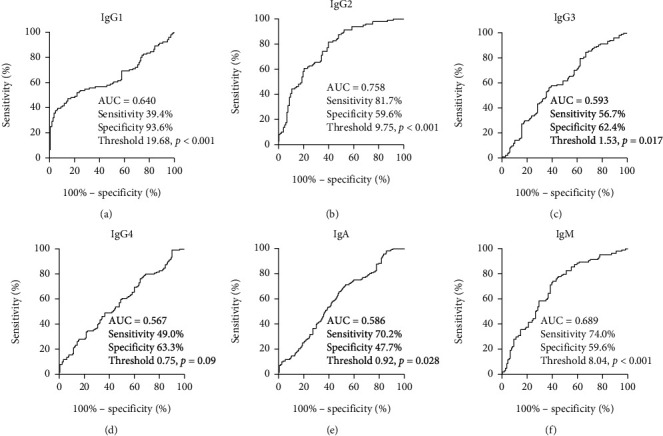
The ROC curve analysis of immunoglobulin test results between CWP and DEW. The ROC curve analysis was performed for (a) IgG1, (b) IgG2, (c) IgG3, (d) IgG4, (e) IgA, and (f) IgM to obtain the threshold values, sensitivity, and specificity. ROC: receiver operating characteristic. CWP: coal workers' pneumoconiosis; DEWs: dust-exposed workers; HCs: healthy controls.

As healthy people have no history of dust exposure and will not be diagnosed as pneumoconiosis, only CWPs and DEWs were compared. To identify the optimal cut-off for the immunoglobulin, the ROC curve analysis was used to evaluate the discriminatory power (Figure 2). The order of diagnostic accuracy by the ROC curve was IgG2, IgM, IgG1, IgG3, and IgA. The AUC for IgG2 was 0.758 (95% CI 0.694-0.814, *P* < 0.01) with an 81.7% sensitivity and 59.6% specificity for discrimination CWP, and the threshold value was 9.75 g/L. The AUC for IgM was 0.689 (sensitivity 74.0%, specificity 76.9%), for IgG1 was 0.64 (sensitivity 39.4%, specificity 93.6%), for IgG3 was 0.593 (sensitivity 56.7%, specificity 62.4%), and for IgA was 0.567 (sensitivity 70.2%, specificity 47.7%).

### 3.4. Validation of the IgG2/IgG3 Ratio for the Diagnosis of CWP

**Figure 3:**
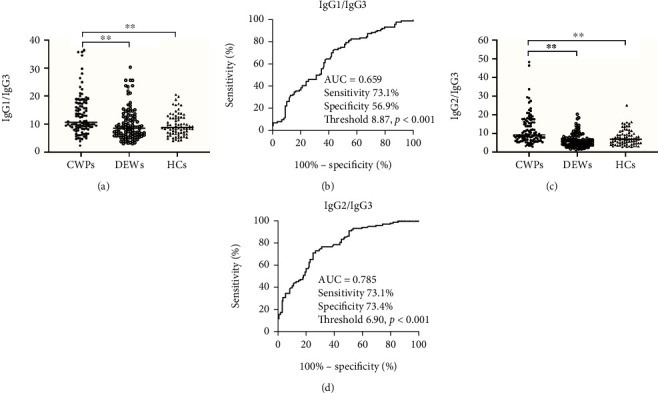
Comparison of the IgG1/IgG3 ratio and IgG2/IgG3 ratio among the study groups. (a) IgG1/IgG3. (b) ROC analysis was performed for IgG1/IgG3 to determine the threshold value for differentiating between CWPs and DEWs. (c) IgG2/IgG3. (d) ROC analysis was performed for the E IgG2/IgG3 ratios to determine the threshold values for discriminating between CWPs and DEWs. ^∗∗^*P* < 0.01. CWP: coal workers' pneumoconiosis; DEWs: dust-exposed workers. ROC: receiver operating characteristic.

We then reviewed serum IgG subclass levels in participants. Unlike the other subclasses of IgG, the concentration of IgG3 of CWPs was lower than that of DEWs and HCs. To further analyze the role of IgG subclass in discriminating CWP from DEW, we proposed a new method to calculate IgG1/IgG3 and IgG2/IgG3. Significantly higher IgG1/IgG3 and IgG2/IgG3 were observed in the CWP group than in DEW and HC groups (Figures 3(a)–3(c)). The performance of the IgG1/IgG3 and IgG2/IgG3 was superior to the AUC of IgG1 and IgG2 alone. The optimal AUC was obtained using the IgG2/IgG3. The AUC of the IgG2/IgG3 ratio was 0.785 (95% CI 0.723-0.838, sensitivity 73.1%, specificity 73.4%), with a threshold value of 6.90 (Figure 3(d)).

### 3.5. Spirometry Test

**Figure 4:**
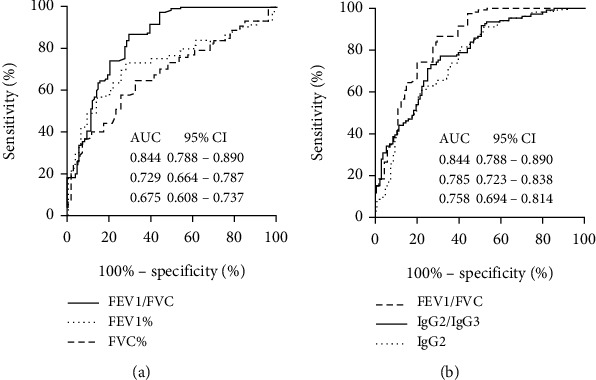
ROC analysis of the spirometry parameters and the IgG2/IgG3 ratio for distinguishing CWPs and DEWs. (a) ROC analysis of the spirometry parameters for CWP and DEW discrimination. (b) ROC analysis of the IgG2/IgG3 ratio, FEV1/FVC, and IgG2 for CWP and DEW discrimination. FVC: forced vital capacity; FEV1: forced expiratory volume in the first second; AUC: area under the curve.

The AUC for FEV1/FVC was larger than that for FEV1 and FVC (Figure 4(a)). The AUC for FEV1/FVC was 0.844 (95% CI 0.788-0.890, *P* < 0.01) with an 86.5% sensitivity and 70.6% specificity for discrimination CWP, and the threshold value was 78.17%. The AUC of IgG2/IgG3, IgG2, and FEV1/FVC was compared using the Delong test (Figure 4(b)). The AUC of IgG2/IgG3 was similar to that of IgG2 (*P* = 0.30), and FEV1/FVC had a better performance compared to IgG2 alone (*P* = 0.04), but no difference to IgG2/IgG3 (*P* = 0.14).

## 4. Discussion

The present study showed increased serum IgG1 and IgG2 levels, decreased IgG3 levels, and relatively static IgG4 levels in the CWP group. This is the first study to our knowledge to quantify IgG subclasses in CWPs. We also reported a novel method calculated from IgG2/IgG3 to distinguish CWPs and DEWs. The IgG2/IgG3 was superior to IgG2 along but was similar to FEV1/FVC.

Currently, international respiratory surveillance includes health and exposure questionnaires, spirometry tests, X-rays, and high-resolution computed tomography. Globally, chest X-rays are typically employed for lung disease diagnosis according to the International Labour Organization (ILO) Classification System [11]. However, diagnostic outcomes of pneumoconiosis by chest X-rays are unsatisfactory [13]. Spirometry tests contribute to the diagnosis and monitoring of pneumoconiosis [14–16]. In our study, FEV1/FVC yielded the best performance in distinguishing CWP and DEW compared to FEV1% and FVC%. This was in line with the result from another study in Chinese coal miners [17], in which the AUC was 0.741 with sensitivity 74.8% and specificity 67.3%. In the present study, it had a larger AUC 0.844, with a sensitivity of 86.5% and a specificity of 70.6%. However, spirometry tests cannot distinguish pneumoconiosis from COPD, and the practical implications and inconsistencies in performing the test need further investigations.

There is substantial evidence supporting that the inhalation of coal dust triggers immune reactions [18, 19]. Coal dust can directly or indirectly induce B-cell proliferation [20, 21]. It showed that draining lung-associated lymph nodes were the most important sites for increased IgG and IgM production in experimental silicosis [22]. Previous studies have shown that increased IgA and IgG concentration in CWP patients while IgM concentration was not significantly different [4, 23]. As these findings were reported from European countries and using the radial immunodiffusion technique, we first verified levels of IgA, and IgM in Chinese coal mine workers. Our data showed that serum IgA concentration increased and IgM also elevated. Increasing of IgM was commonly reported in silicosis or silica dust contacted [24–26], so we speculated the discrepancy may be due to differences between coal mines and, thus, the coal dust composition or different genetic background [27–29].

We quantified the IgG subclass instead of IgG and showed concentration changes of IgG1, IgG2, and IgG3. To our knowledge, this is the first time the IgG subclass has been detected in patients with coal mine pneumoconiosis. A study has demonstrated that a subcutaneous injection of carbon black in female BALB/C mice increased the IgG1 and IgG2a serum concentration while a silica injection predominantly increased IgG2a [30]. In human, IgG subclasses are involved in several autoimmune disorders such as rheumatoid arthritis, primary Sjogren's syndrome, systemic sclerosis, systemic lupus erythematosus, and primary biliary cirrhosis [9, 31]. Coal mine dust can also cause autoimmune disease like Caplan's syndrome [32]. Therefore, it is not surprising that CWP and connective tissue disease-associated interstitial lung diseases share similar features of hyperinflammation, collagen deposition, and lung fibrosis [33, 34].

The ROC curve analysis showed that the AUC was the largest for IgG of the immunoglobulins we examined, indicating that it is the most accurate one for the diagnosis of CWP. We also noticed that IgG3 declined which was different from the other immunoglobulins; therefore, we propose a new calculation method for estimating the ratios of IgG1 and IgG2 to IgG3. We found that the IgG2/IgG3 ratio or the IgG1/IgG3 ratio had larger AUC than the single-factor diagnoses. We then compare the IgG2/IgG3 ratio to FEV1/FVC and found that although IgG2/IgG3 had no difference to IgG2 along (*P* = 0.30), the AUROC of FEV1/FVC had a better performance compared to IgG2 alone (*P* = 0.04), but no difference to IgG2/IgG3 (*P* = 0.14). Consider the limitation of spirometry to diagnose CWP we talked above; these data suggest that the IgG2/IgG3 ratio could be used to differentiate between CWP and DEW.

Certain limitations should be considered in this study. First, we did not test inflammatory cytokines the same time; our data do not allow us to address the underlying mechanisms of immunoglobulins in CWP. In addition, serum immunoglobulin levels may be influenced by genetic background and dust composition; further validation by other centers with a large sample size is required.

## 5. Conclusion

The IgG2/IgG3 ratio could be used as an adjunct tool for diagnosis and exclusion of CWP. However, as this is a single-center study with limitations, an additional calculation method using immunoglobulins needs further validation before it can be used to differentiate CWP from healthy workers.

## Figures and Tables

**Table 1:** Participants' characteristics.

	CWPs (*n* = 104)	DEWs (*n* = 109)	HCs (*n* = 74)	*P* value
Age (years)	58.1 ± 6.6	58.0 ± 7.0	56.6 ± 6.9	0.273
BMI (kg/m^2^)	23.7 ± 3.2	23.9 ± 3.2	24.5 ± 4.0	0.635
Smokers (*n*, %)	81 (77.88%)	77 (70.64%)	57 (77.03%)	0.642
Drinking status (*n*, %)	13 (12.50%)^#^^∗^	30 (27.52%)	14 (18.92%)	0.022
Hypertension (*n*, %)	16 (15.38%)	12 (11.01%)	8 (10.81%)	0.548
White blood cells, ×10^9^/mL	6.5 (5.4, 7.9)	6.0 (5.4, 7.2) ^#^	6.8 (5.8, 8.1)	0.031
Neutrophils, ×10^9^/mL	3.9 (3.2, 5.2)	3.6 (2.9, 4.3)	3.9 (3.5, 4.8)	0.022
Lymphocytes, ×10^9^/mL	1.7 (1.4, 2.2)	1.9 (1.5, 2.3)	2.1 (1.7, 2.6) ^#^	0.005
Exposure time, years	28 (20, 30)	24 (22, 26)	NA	0.290
FVC, predicted %	85.2 ± 17.9^#^^∗^	95.2 ± 13.1	97 ± 14.1	<0.001
FEV1, predicted %	72.2 ± 24.0^#^^∗^	88.4 ± 13.7	92 ± 14.0	<0.001
FEV1/FVC, %	68.8 (58.0, 76.3)^#^^∗^	84.2 (76.2, 88.2) ^#^	75.3 (72.1, 79.0)	<0.001

*P* values were calculated by the chi-square test, Kruskal–Wallis test, one-way analysis, Mann–Whitney *U* test, as appropriate. BMI: body mass index; FVC: forced vital capacity; FEV1: forced expired volume in the first second. ^#^Compared with healthy controls, adjusted *P* < 0.05. ^∗^Compared with DEWs, adjusted *P* < 0.05.

## Data Availability

The datasets used during the present study are available from the corresponding author upon reasonable request.
